# Alcohol and Exaggeration

**Published:** 1908-09-12

**Authors:** 


### The Hospital

A Journal of

The Medical Sciences and Hospital Administration.

New Series. No. 79, Vol. III. [No. 1151, Vol. XLIV.] SATURDAY, SEPTEMBER 12, 1908.

### ALCOHOL AND EXAGGERATION.

In a recent issue of the *Medical Record* of New York a correspondent gives a brief history of the American Society for the Study of Inebriety and Alcoholism, with a *précis* of the proceedings at its last annual meeting. As far as may be judged from this *précis*, the meeting produced little that is novel. The communications, which were many, were for the most part confined to a comprehensive abuse of alcohol. It is perhaps not unnatural that those whose lives are devoted to remedying the mischiefs wrought by alcohol should find no good word to say for a drug so manifestly responsible for human tragedies. Nevertheless, we cannot afford to be content with *ex parte* statements, and should have been grateful for some recognition of the fact that the use and the abuse of alcohol are by no means synonymous terms. We are not ourselves disposed —human nature being what it is—to defend the use of alcohol as a beverage purely on its merits, and are ready to believe that its total elimination from the dietary of mankind would, upon balance, be an advantage to the race. But since such a total elimination is outside the sphere of practical economics for many a long year to come, we must, if we wish to arrive at the truth, attempt at least to state the position fairly, and separate as rigidly as possible the evil consequences, if any, attending the use of the drug from those which bear such ample evidences to its abuse. This is an old theme and much argued, but the need for clear thinking upon it is still necessary, for there is hardly a problem of all those that face our civilisation around which bias and prejudice grow so luxuriantly as this. Nothing is so fatal to the success of a cause as over-statements in the mouths of its propagandists. This, at least, is true of this country, where fortunately the majority, though liable to gusts of temporary unreason, is in the main remarkably level-headed and capable of detecting and despising exaggeration and windy hyperbole. If we appear to join issue upon some points with the ardent school of temperance, it is not because we yield to anyone in the wish to see our countrymen temperate, but because in the long run over-statement brings its own punishment, and we would not willingly risk a set-back to the cause of sobriety.

Let us, then, consider in the first place what evidence there is that alcohol taken in moderation is the noxious substance we are asked to believe it. We are, of course, met at once by the difficulty of defining moderation, and there are no doubt scores of men who habitually take more alcohol than is good for them who nevertheless consider themselves moderate drinkers because they are never drunk. Still, it is not necessary to define very precisely what is true moderation, for any fair-minded reader will have to confess that the majority of his acquaintances furnish representatives of that vague but common type—the moderate drinker. It is probable that the generic condemnation of alcohol in all forms is based upon laboratory observation. Thus, one of the speakers at the meeting mentioned above is reported to have said "that laboratory research work from every point of view confirmed the statement that alcohol was a paralysant, and its action on the cells and tissues was corroding and destructive, both chemically and physiologically." This is no doubt true. A substance which is in common use for the "fixing" of tissues—that is to say, for the coagulation of the albumin in them, is *ex hypothesi* damaging to a living structure. Yet it does not follow that, because alcohol in strong solution is so damaging to living things as to be a recognised preservative against putrefaction; therefore, in the diluted form in which it is employed by moderate drinkers it is still noxious. As well might one say that because a strong mustard poultice will take the skin off a man's back, therefore the mustard which he takes with his bacon will destroy his gastric mucous membrane. Nor is it any more just to say that, because the exhibition of alcohol in considerable doses to an animal totally unaccustomed to it is capable of producing degeneration of the liver; therefore, a glass of wine for dinner will exercise the same effect upon a man well accustomed to it. These fallacies of deduction, based upon laboratory findings, when thus stated in precise terms seem so platitudinous as almost to demand an apology for their insertion in this place; yet they form the often-repeated text of much random sermonising about alcohol and its ill-doings. As a matter of fact, there is no need for laboratory findings, for the human experiment is perpetually before us. On the one hand we have in abundance the healthy, hard-working business man who throughout a long and arduous life seldom misses his glass or two of wine with lunch and dinner, and dies in or about the seventies without having lost either his good name or his digestive powers. It is idle to affirm that this man has poisoned himself, though we are content to yield to the extreme faction the possibility that had he abstained altogether he might have lived to be 80. On the other hand we have the equally convincing experiment afforded by the sodden toper who dies an alcoholic dement at 40. It seems to us that the facts leave no room for the statement that alcoholic beverages are inherently poisonous, and to affirm that they are, without qualification, is to weaken by an untruth the excellent case which can be made for sobriety. While deploring as sincerely as any the curse which some men make of alcohol, not only for themselves but for their dependents and society in general, we are not prepared to shut our eyes to its value in the promotion of good fellowship among those who have more self-control. Enthusiastic advocates of temperance will do more for their cause by urging the demonstrable evils of excess, than by going beyond their brief at the risk of disgusting moderate opinion.

